# Statistical Approach of Functional Profiling for a Microbial Community

**DOI:** 10.1371/journal.pone.0106588

**Published:** 2014-09-08

**Authors:** Lingling An, Nauromal Pookhao, Hongmei Jiang, Jiannong Xu

**Affiliations:** 1 Department of Agricultural & Biosystems Engineering, University of Arizona, Tucson, Arizona, United States of America; 2 Interdisciplinary Programs in Statistics, University of Arizona, Tucson, Arizona, United States of America; 3 Department of Statistics, Northwestern University, Evanston, Illinois, United States of America; 4 Department of Biology, New Mexico State University, Las Cruces, New Mexico, United States of America; Indiana University, United States of America

## Abstract

**Background:**

Metagenomics is a relatively new but fast growing field within environmental biology and medical sciences. It enables researchers to understand the diversity of microbes, their functions, cooperation, and evolution in a particular ecosystem. Traditional methods in genomics and microbiology are not efficient in capturing the structure of the microbial community in an environment. Nowadays, high-throughput next-generation sequencing technologies are powerfully driving the metagenomic studies. However, there is an urgent need to develop efficient statistical methods and computational algorithms to rapidly analyze the massive metagenomic short sequencing data and to accurately detect the features/functions present in the microbial community. Although several issues about functions of metagenomes at pathways or subsystems level have been investigated, there is a lack of studies focusing on functional analysis at a low level of a hierarchical functional tree, such as SEED subsystem tree.

**Results:**

A two-step statistical procedure (metaFunction) is proposed to detect all possible functional roles at the low level from a metagenomic sample/community. In the first step a statistical mixture model is proposed at the base of gene codons to estimate the abundances for the candidate functional roles, with sequencing error being considered. As a gene could be involved in multiple biological processes the functional assignment is therefore adjusted by utilizing an error distribution in the second step. The performance of the proposed procedure is evaluated through comprehensive simulation studies. Compared with other existing methods in metagenomic functional analysis the new approach is more accurate in assigning reads to functional roles, and therefore at more general levels. The method is also employed to analyze two real data sets.

**Conclusions:**

metaFunction is a powerful tool in accurate profiling functions in a metagenomic sample.

## Introduction

Metagenomics is the study of genetic material recovered directly from natural (e.g., soil or seawater) or host-associated (e.g., human gut) environmental samples that contain microorganisms organized into communities. The advancement of high-throughput next generation sequencing technologies provides a powerful way in metagenomic studies since they can be directly applied to an environmental sample without the need of isolating and culturing individual microbial species in a laboratory. More than 99% of millions microbial species on Earth cannot be cultured in a laboratory [1,2]. The massively parallel sequencing technologies, such as 454FLX, Illumina Genome Analyzer (GA), and ABI SOLiD, have enabled us to generate millions of reads (35-500 base pairs (bp), depending on the platform) at a time [3]

The initial computational analysis of metagenomics focuses on two main questions: who is out there and what they can do [1,2]. To answer the first question, scientists determine taxonomic compositions in a particular metagenomic sample and determine the abundance/proportions of the species. Many methods have been proposed [4–7], particularly, TAMER8], GASSiC [9], and TAEC [10] focus on the taxonamic analysis at a very low phylogentic level - species.

**Figure 1:**
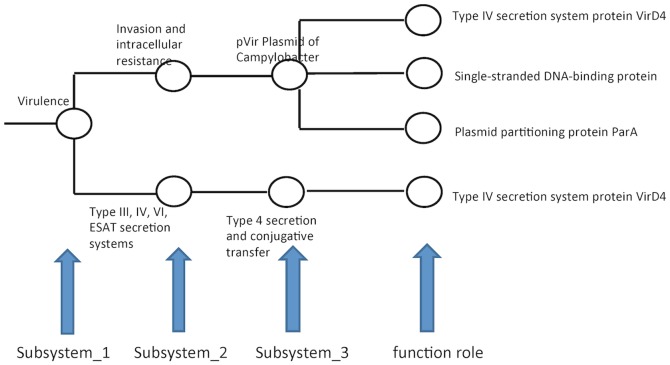
Illustration of subsystem tree structure in SEED.

To answer the question “what they can do” scientists need to determine the gene contents, functional categories, and estimate the relative functional abundances contributed in the metagenomic sample. According to Overbeek et al. [11], a functional role corresponds roughly to a single logical role that a gene or gene product may play in the operation of a cell, such as ‘Aspartokinase (EC 2.7.2.4)’, and pathway or subsystem which is a collection of related functional roles (Figure 1). To characterize the functional capacity of a metagenomic community, therefore, researchers can perform analysis either at the functional role level or pathways/subsystems level. Most recently published studies focused on pathways or subsystems level [12–15]. However, a number of questions about functional roles of microbial communities are still ambiguous, e.g., do microbial communities consist of extensive genetic diversity, how are they diverse in functional roles, how does the diversity in functional roles of microbial communities affect their interaction with environment? Performing function analysis of metagenomes at functional roles level, therefore, is an appropriate approach to addressing these issues. Through such type of analysis, functional roles can be detected and further metabolic pathways or subsystems that the functional roles are involved can be established [14].

Several tools have been developed to detect/annotate functional roles from a metagenomic sample [16]. Among the commonly used publicly available pipelines, most of them are homology-based tools, such as MEGAN [17], MG-RAST [18], IMG/M [19], and CAMERA [20]. In MEGAN the functional analysis of metagenomes is based on the SEED hierarchy [18]. The SEED has consistent and accurate microbial genome annotations of any publicly available source [11]. To perform a functional analysis, MEGAN assigns each read to the functional role of the highest scoring gene in a BLAST comparison against a protein database (e.g., NCBI-NR), and then different functional roles are grouped into SEED subsystems. The SEED classification can be represented by a hierarchical tree, where the internal nodes represent subsystems and the leaves denote the functional roles (Figure 1).

However the MEGAN program has several disadvantages. First of all, the best score assignment might miss putative functions. Because of the existence of sequencing error [21], a sequence read could come from a gene/function with aligned matches of 32 out of 33 codons and could also from a gene/function with aligned match of 31 out of 33 codons. The MEGAN method misses the second or even the third best scoring functions that the read may have. Furthermore, a gene could play multiple functions at the same time. However MEGAN just assigns one function (with the best match value) to the short read even when multiple functions show the same best match values (e.g., the e-value, bitscore, or the number of matched codons). For example, blastx output for a short read shows two functions “*Argininosuccinate lyase (EC 4.3.2.1)*” and “*N-acetylglutamate synthase (EC 2.3.1.1)*” with the same best match values, but MEGAN only assigns the first function (alphabetically) to the read. Thus, MEGAN misses some functions existing in the community and therefore underestimates their abundance.

MG-RAST [18] can assign multiple functions to a read, but some flat cutoffs, e.g., e-value < 1.0e-5 and identity cutoff > 60% are used. Thus assignment of reads to different ranks of taxonomy tree greatly depends on the threshold of bit-score or Expect value used. As a consequence, the results lack specificity. IMG/M uses the best BLAST hits for function assignment [19]. In CAMER [20] open reading frames (ORFs) are clustered at a certain cutoff of identity (e.g., 60%) over a certain threshold (e.g., 80%) of ORF length. ORF clusters are then used for functional studies. Both the best-hit approach (in MEGAN and IMG/M) and objective cutoff approach (in MG-RAST and CAMER) lack of statistical support.

Motivated by both the advantages and limitations of these methods and inspired by the statistical model in Jiang et al. [8] we propose a two-step procedure to accurately assign functions to reads. In the first step sequencing error is estimated through a mixture model, which is proposed to model the translated sequence reads at the base of codons and detect the possible functions in a metagenomic sample. As a gene could be involved in multiple biological processes, the functional assignment is adjusted by utilizing an error distribution at the second step. The proposed two-step method is comprehensively tested on simulated metagenomic data with diverse complexity of microbial community structure, and also applied on two real metagenomic datasets. Compared with MEGAN and MG-RAST for functional metagenomic analysis, the proposed approach demonstrates greater accuracy in function identification and abundance quantification. The R package “metaFunction” is available for download at http://cals.arizona.edu/~anling/software.htm.

## Methods

**Figure 2:**
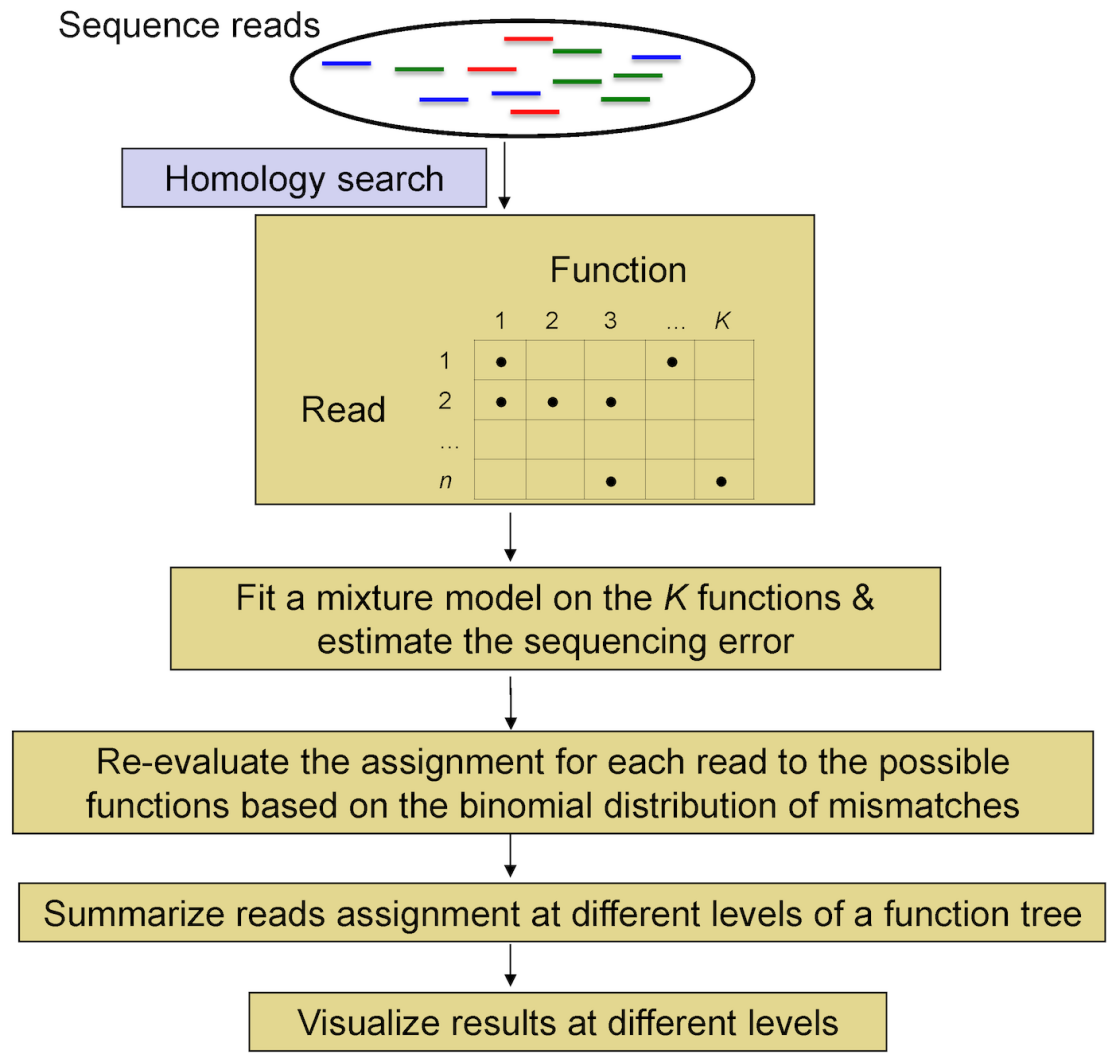
Flowchart of the proposed method - metaFunction.

For each sequence dataset we use BLASTX to search for matched reference sequences (i.e., genes) in the NCBI-NR protein database. Then genes are classified into functional role categories as defined by the SEED classification. Based on the sequence reads we need to estimate: (1) the sequencing error rate and (2) functional roles contained in the metagenomic sample and their relative abundance (i.e., proportions). To answer these questions, we set up a mixture model based on the information from BLASTX results. And then a binomial model for the sequencing error (estimated by the mixture model) is proposed to adjust the function assignment, and therefore the proportion estimation for each function is adjusted accordingly. The adjustment on assigning functions is to incorporate the fact that a gene/short read could play multiple function roles. The flowchart for the proposed procedure can be found in Figure 2.

### Estimate sequencing error

Suppose we have *n* sequence reads that are mapped to sequence homologs in the reference database (i.e., NR protein database) and return *K* functions (i.e., gene families) in the result of homolog research, e.g., BLASTX output. Let 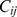
denote the number of identical matched codons for read *i* under functional role *j* and 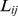 represent the corresponding aligned codon length. Let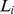 denote the maximum aligned codon length for read *i* across all candidate functions, i.e., 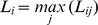 then we have 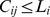. If the read *i* does not have matched sequences for function *j*, then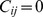. We assume that the larger the 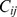
value, the more likely that the read *i* performs function *j*. Let 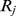 denote the proportion of reads having function *j*, thus 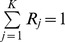. Even if the read *i* is from function *j*, it is also possible that 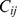
is not exactly as same as 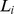, the maximum aligned length. It may be due to the sequencing error and/or single nucleotide polymorphism (SNP) effect or various sources (i.e., organisms) for the same gene in the database. Let *p* denote the probability of observing a mismatched codon, then 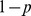 is the probability of observing an identity or conserved codon. Therefore the probability that the read *i* performs function *j* with 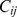 matched codons and 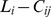
mismatched codons is 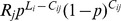. Then the probability to observe the read *i* in the dataset is 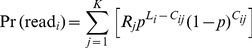
(1)


Hence the likelihood function of the data is: 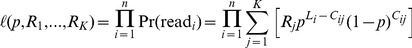
(2)


In this likelihood function, the maximum aligned length 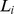 and the matches 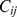
can be extracted from the BLASTX output. The parameters *p* and 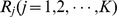 are then estimated by Expectation Maximization (EM) algorithm [22]. As *p* is the probability for observing a mismatched codon, for simplicity, we just call *p* as sequencing error (rate) and a mismatched codon as a mismatch.

### Multiple-function assignment

One read could get involved in multiple functional roles. For read *i*, assume its best mismatch (i.e., minimum number of mismatched codons) across all functions is 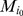, we can determine the maximum allowable mismatch 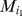 for a given small probability *ε* such that:
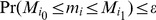
(3)where we assume that the mismatch 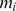 follows a binomial distribution with parameters 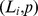. Then read *i* can be assigned to all the functions with mismatch ≤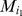. The relative abundance 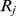
will be updated by this new multiple function role assignment, i.e., the updated one becomes: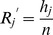, where 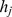
is the number of short reads assigned to the function *j* after the adjustment, and *n* is the total number of short reads in the dataset. Thus we have 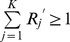. The algorithm procedure for the multiple-function adjustment can be summarized as below:

Based on the estimated sequencing error obtained in step 1 and a pre-specified small probability *ε*:

1. for read *i* calculate its maximum allowable mismatch 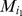 using eq. (3)
2. assign all functions with corresponding mismatch ≤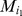 to read *i*
3. repeat steps 1) and 2) for all reads
4. calculate the new roportion 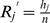 for each function based on these new assignments.

**Figure 3:**
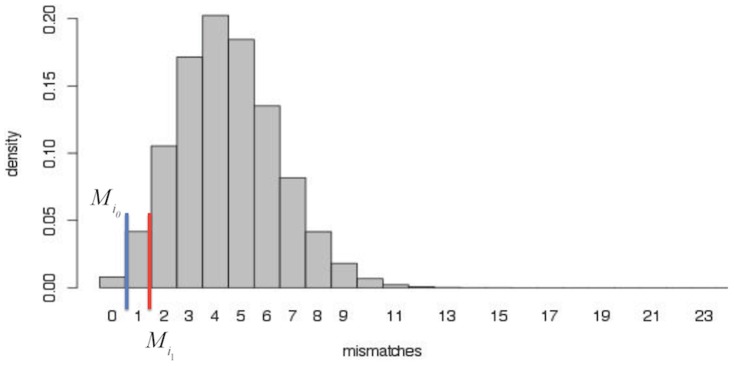
Illustration of calculation of multiple function assignment. In this plot *ε* = 0.05 and the binomial distribution has *p* = 0.15 and *L_i_ = *32.

Figure 3 illustrates the calculation for multiple function assignment based on a binomial distribution. In this illustration the maximum length of the aligned codons is 32 and sequencing error at the base of codon is 0.15. If the best mismatch is 0 and the probability *ε* = 0.05, then the maximum allowable mismatch is calculated as 1. It means that the functions with matched codons of 32 or 31 ( = 32-1) in BLASTX output are all possible and therefore the read is finally assigned to these functions. The small probability *ε* in equation (3) is suggested as one third of the sequencing error at the codon level (estimated in the first step) or just the sequencing error at the nucleotide level, if known or given. More details about the selection of *ε* can be found in the simulation studies below.

### Construct statistical inferences

None of the existing methods on functional metagenomic analysis could further assess the uncertainty of the proportion of assigned reads to functions. We propose to use bootstrap method [23] for constructing the confidence intervals for the estimates. We first draw a bootstrap sample by resampling the reads from the original sequence reads with replacement; the relative abundances are estimated using the described two-step procedure for the bootstrap sample. We repeat this resampling/bootstrap for a large number of times, e.g., 1000 times. Then the confidence intervals can be constructed based on these bootstrap estimates. Since we construct the confidence intervals for the abundances of the *K* functions, *R_j_* (*j* = 1,…, *K*) simultaneously, a multiple correction method, e.g., Bonferroni method [24], is applied to guarantee a pre-specified family-wise confidence level.

### Simulation studies

#### Experimental data

Due to the complexity of metagenomic data, simulation studies with verifiable structure are crucial to benchmark the proposed approach and to conduct comparisons with other existing methods. So far there is no literature about how to set up a simulation study for functional metagenomics. We propose to use the SEED database (http://pseed.theseed.org) and conduct six different simulation studies. Basic information of these six simulation settings is listed in Table 1. Similar to the studies in MetaSim [25] which contain a small number of genomes in each setting we simulate a small number of functions in each study.

**Table 1:** Basic information of six simulation studies.

Study	Characteristic of the 10 primary functional roles	Sampling rate from SEED database
1	Different	fixed 20%
2	Different	20∼40%
3	Closely related	fixed 20%
4	Closely related	20∼40%
5	Same as study 1 & 2	Large sample size
6	Same as study 3 & 4	Large sample size

Study 1 contains 10 function roles that are far away from each other in the SEED tree. For each function role, 20% of the sequences (i.e., FIGfams, very long sequences) from the SEED database are chosen and the sampling rate for this situation is 20%. Then a short segment of 100 bp is randomly chopped from the selected long sequence, and 2% sequencing error is added to it. The sequencing error could be due to the substitution, deletion and insertion. For the purpose of method illustration we only consider the substitution error. It is well know that some genes are involved in multiple functions in a microbial community. This is also reflected from the gene sequences in the SEED database, i.e., some long sequences are labeled with multiple functions. As expected, a few additional function names are obtained for the short reads in the 10 pre-selected groups. We name them secondary functions, and the 10 pre-selected functions as primary functions (see the Table S1 in File S1). The number of short reads for each function is also listed in the table S1 in File S1. Both types of functions are treated as true functions since the functions in either type are the true ones for the generated short reads.

Study 2 contains the same 10 primary functions as study 1 but with various sampling rate (see the Table S1 in File S1). The number of short sequence reads generated for each function is based on the total number of long sequences in the function group in the SEED database. Generally, the sampling rate varies between 20%∼40%. In studies 3 and 4 we use another set of 10 functions (see the Table S2 in File S1). Different from the studies 1 and 2, the 10 function groups here are very closely related (i.e., some functional roles are belong to the same subsystems). Study 5 contains the same 10 primary function groups as studies 1 & 2 but the sampling rate is much larger, about 4∼5 times of the first two studies; similarly, study 6 contains the same 10 primary function groups as studies 3 & 4 but the sampling rate is about 4∼5 times of these two studies (see the Table S1 and S2 in File S1). The coverage, i.e., ratio of the number of simulated base pairs to the total number of base pairs for the selected functions in the SEED database, varies 2%∼9% in these six studies.

#### Simulation Results

**Figure 4:**
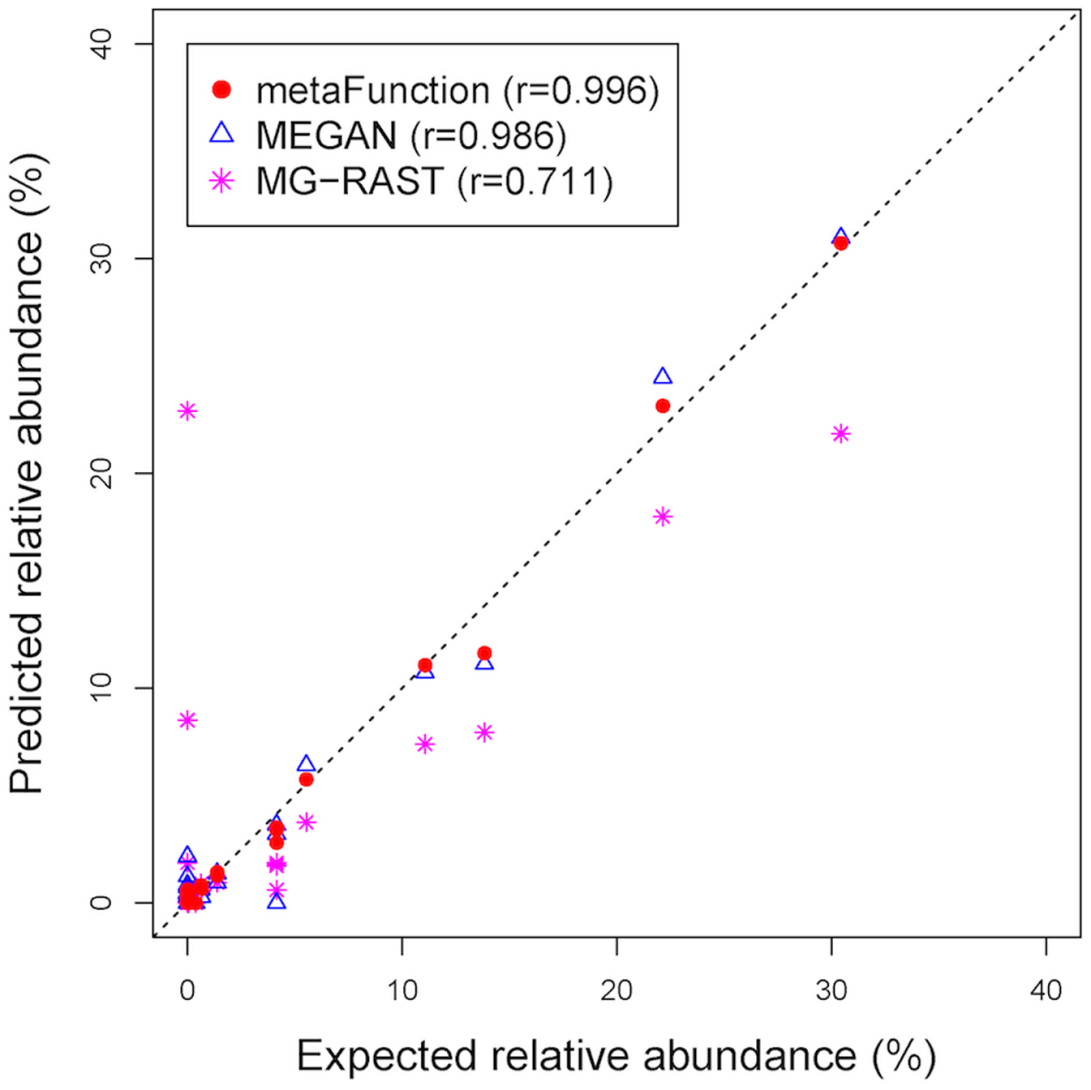
Scatter plot of the predicted vs. expected (true) relative abundance of the functions in Simulation 1.

Three methods, MEGAN (best hit), MG-RAST (flat cutoff) and the proposed method metaFunction, are compared through these six simulation studies. The result for the first simulation study is shown in Figure 4 where it plots the relationship between the estimated (i.e., predicted) abundance for each function and its true (i.e., expected) abundance. If all the functions are detected and their abundances are correctly estimated then the Pearson correlation between the expected and predicted abundances is one. From the plot it is obvious that the proposed approach has the largest correlation. Table 2 displays the summary of the correlations in all six studies for these three methods. The new method outperforms the other two methods in all studies in terms of correlation between the true and estimated abundances. While MEGAN and metaFunction methods perform better on the distant function studies (studies 1, 2 and 5) than on the closely related function ones (studies 3, 4 and 6), MG-RAST seems work better on the closely related functions. It is because in the distant function studies MG-RAST detects a false function “decarboxylase” with a very large proportion. This greatly affects the correlation calculation.

**Table 2:** Summary of the correlation values in all six studies by three methods.

	Study 1	Study 2	Study 3	Study 4	Study 5	Study 6
MEGAN	0.986	0.968	0.852	0.839	0.973	0.917
MG-RAST	0.711	0.696	0.880	0.857	0.750	0.895
metaFunction	0.996	0.993	0.953	0.943	0.997	0.982

The correlation is calculated between the expected (i.e., true) and estimated abundance for the simulated functions.

**Figure 5:**
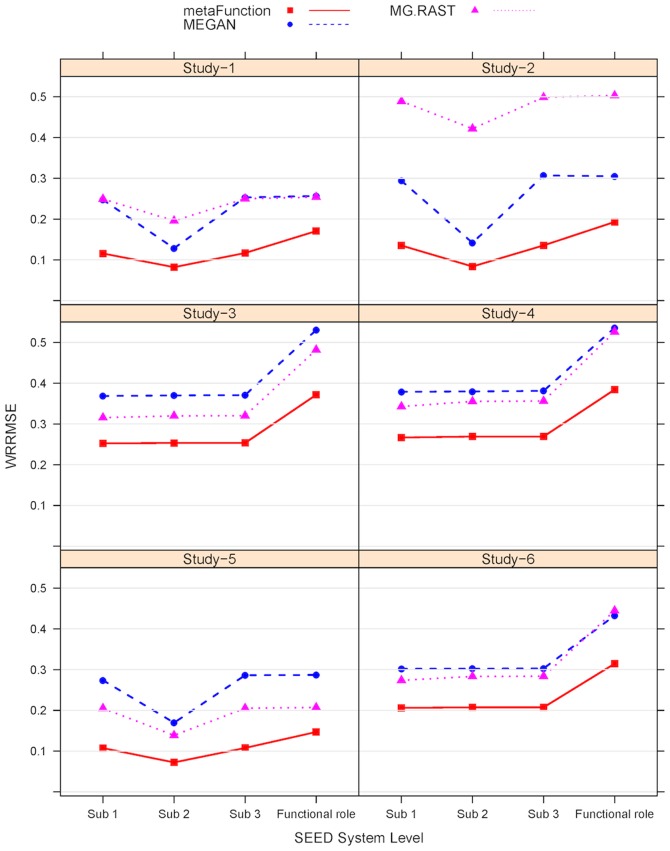
Plot of WRRMSE values for three methods and in six simulation studies. Weighted Root of Mean Square Relative Error (WRRMSE) is calculated between the true function/subsystem and the estimated function/subsytem by each method (MEGAN, MG-RAST, and metaFunction).

We also evaluate the performance of three methods via the same simulations using another metric. A common measure for error is root mean square of relative error [7,26]. In this definition each feature group is assumed the same weight in the error calculation, regardless the abundance of features in each group. In function analysis of metagenomics a function group estimated with a tiny number of counts actually should much less likely exist in the sample than a group with large number of read counts. We modify the error measure to weighted root mean square of relative error (WRRMSE), i.e.,


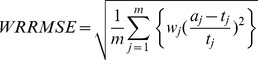
, where the weight 
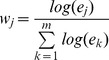
,


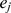
 is the estimated number of reads for function *j*, 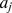 is the estimated relative abundance (i.e., estimated proportion) and 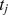 is the true relative abundance, and *m* is the number of true function groups. The WRRMSE results for six studies are shown in Figure 5. In each of subplots the x-axis is the SEED system level. Compared to the MEGAN and MG-RAST, the proposed method has the lowest error at any level of the subsystems and for all simulation studies. Decrease in Error on Sub 2 level in Figure 5 is due to the unnamed subsystems in the SEED tree. For example, a read is assigned to a level-3 subsystem but its parent node has no name (i.e., NULL) then the assignment to this unknown level-2 subsystem will be excluded in calculating the error. The decrease in error for sub 2 level is due to the removal of the NULL group that may contain some wrong assignments.

The accuracy on estimation of relative abundance plays an important role in metagenomic analysis, the accuracy of assignment of short reads is also very interesting to biologists in functional metagenomics as they need the information of what reads do what kind of functions. As the MG-RAST does not give the information of the assignment we compare the performance of MEGAN and the proposed method metaFunction regarding the assignment details. In each of six simulation studies we calculate the proportion of correctly assigned (CA), wrongly assigned (WA), and not assigned (NA, i.e., not aligned to the reference database) across all functions. The assignment details are also examined at other levels of the subsystem. The results of the simulation study 1 are displayed in Table 3. At any level of the subsystems (including the function level) the proportions of NA using metaFunction are lower than those from the MEGAN result. The WAs for metaFunction are little higher than the ones for the MEGAN but they are comparable (all <1%). The new approach results in much higher CA rate than MEGAN (about 90% vs 70%). Consistent conclusions are obtained for other simulation studies (data not shown).

**Table 3:** Proportion of correctly assigned (CA), wrongly assigned (WA), and not assigned (NA) simulated reads by MEGAN and metaFunction.

	MEGAN			metaFunction		
	CA (%)	WA (%)	NA (%)	CA(%)	WA (%)	NA (%)
Function	77.45	0.22	22.55	91.06	0.39	8.94
Subsystem 3	77.22	0.22	22.78	91.05	0.38	8.95
Subsystem 2	76.66	0.21	23.34	91.11	0.37	8.89
Subsystem 1	76.66	0.21	23.34	91.11	0.37	8.89

This result is for different levels of the SEED tree in the first simulation study.

#### Selection of ε

The above results are based on 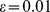 in eq. (3). We conduct another study to investigate the effect of selecting different small probability *ε* on the final result in terms of the error metric defined above. Let *ε* take various values of 0.01, 0.05, or 0.1 for the multiple role assignment. Within each of the above six simulated experiments the WRRMSE values are very close for these three different *ε* values. That is, the absolute difference on WRRMSE is less than 0.0001 between the two situations of *ε* = 0.01 and *ε* = 0.05; and less than 0.008 between *ε* = 0.01 and *ε* = 0.10. In terms of relative difference on WRRMSE, the values are (0∼2%) for different *ε.* Therefore, the final result is not sensitive to the selection of *ε*. In the above six simulated experiments 2% error is added to each short read, any value between 0.01 and 0.05 is plausible for *ε.*

## Real Data Analysis

Real metagenomic data from an environmental study and a human health study are analyzed using the proposed method - metaFunction.

### Environmental study

Metagenomic functions were compared between Lake Erie (North America) and Lake Taihu (China) [27]. Toxic *cyanobacteria* blooms appear to be a global problem as toxins produced by bloom-associated *cyanobacteria* can have drastic impacts on the ecosystem and surrounding communities; in addition, the produced bloom biomass can disrupt aquatic food webs and act as a driver for hypoxia. Freshwater samples were collected from different lakes to examine the bloom associated microbial communities. We select two lakes - Lake Erie and Lake Taihu as they represent different continents – to examine the gene contents. After quality checking totally 750 thousands reads with an average length of 425 bp are aligned to the NCBI non-redundant database. Then the proposed method is applied to the alignment output. The original study used both MEGAN and MG-RAST for functional annotation and they addressed that the two results are highly consistent. We compare our result to the MG-RAST result in the original paper, which are downloaded from the MG-RAST online server (http://metagenomics.anl.gov/) under the identification numbers 4467029.3 (Erie), 4467058.3 (Taihu).

**Figure 6:**
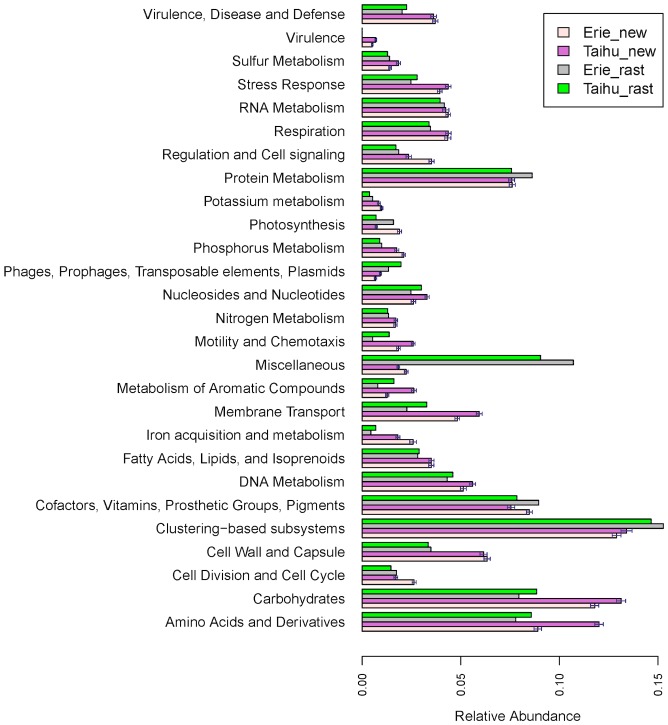
Proportions of the detected subsystems (level 1) by MG-RAST and metaFunction for the lake data. The top 27 subsystems with proportion >0.005 in at least one of samples are listed. The “error” bars represent the 95% confidence interval obtained by bootstrap method. Note: only the proposed approach can provide confidence intervals for the estimations of the proportions.

The functionality profiles of microbial communities in these two lakes by metaFunction and MG-RAST are summarized at the level 1 of subsystem (Figure 6). Generally, the results from these two approaches are consistent: the subsystems found by one method with big proportions are also detected by the other with large amount. However there also exists some discrepancy between the two results. The subsystem “Miscellaneous” is found dominant by MG-RAST in both lakes but not ample by the new method; “Virulence, Disease and Defense”, “Virulence”, “Membrane Transport”, and “Cell Wall and Capsule” are observed more abundant by the new approach than by MG-RAST.

When compare the results between two lakes we found that subsystems abundant in one lake by the MG-RAST often show plenty in the same lake by the new method. For instance, “*Amino Acid and Derivatives*”, “*Carbohydrates*”, “*Nucleosides and Nucleotides*”, and “*Membrane Transport*” are found more abundant in Lake Taihu than in Lake Erie. Meanwhile “*Cell Division and Cell Cycle*”, “*Regulation and Cell signaling*”, “*Cofactors, Vitamins, Prosthetic Groups, Pigments*” are lower in Lake Taihu. A big difference tween the results from two approaches is that the new method can provide confidence interval information for the proportion estimation, which is displayed as the small bars in Figure 6. Thus the new method can provide more information about the group comparisons. Comparison between two lakes at a lower level of subsystems - level 3 - is shown in the Figure S1. Not surprising, the results from two approaches in Figure S1 are more disparate than at the higher level of subsystems in Figure 6.

### Human Health study

**Figure 7:**
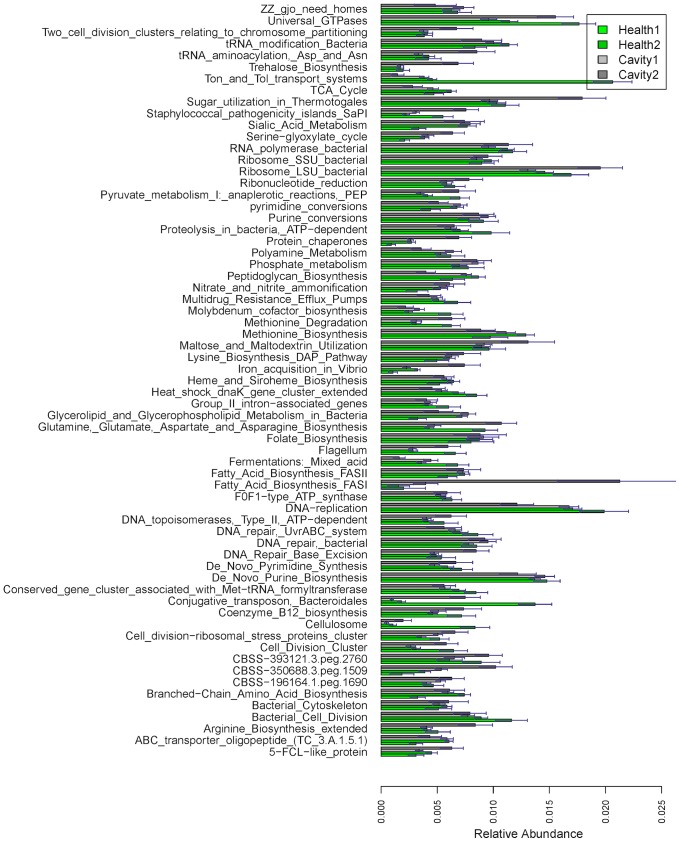
Proportions of the detected subsystems (level 3) for the oral data. The top 78 subsystems with proportion >0.005 in at least one of samples are listed. The “error” bars represent the 95% confidence intervals obtained by bootstrap method.

Human oral microbial samples were studied for oral cavity problem using 454 pyrosequencing [28]. Two healthy samples and two cavity samples are selected for our analysis, with one at an intermediate stage and the other one at an advanced stage of caries development. After quality checking, 0.5 Gbp of sequence with the average read length 425 bp are BLASTXed to NCBI-NR protein database for searching matched reference sequences (i.e., genes). Then reads are classified into functional role categories as defined by the SEED structure using the proposed method. The results of functionality profiling for all four samples at the subsystem level 3 are shown in Figure 7.

In this plot the abundance of “*Conjugative transposon Bacteroidales*” is much higher in the cavity samples than in the healthy orals, which is also confirmed in other literature [29]; “*Fatty Acid Biosynthesis FASI*” also shows a higher value in the diseased samples than in the healthy samples, which is consistent with the finding in [30]; That the “*Flagellum*” is abundant in the cavity samples is also reported in Seshadri et al. [31]; high values of “*Glutamine Glutamate, Aspartate and Asparagine Biosynthesis*” and of “*Methionine degradation*” in the oral cavity samples are also mentioned in other publications [32,33]; the abundance of “*Universal GTPases*” is higher in the cavity samples than in the healthy orals, which is also found in other literature [34]. In conclusion, the results from the new method provide us the findings consistent with the previous literatures.

## Discussion

One of the main challenges in metagenomic studies is how to accurately identify all possible functional roles present in an environmental sample and precisely estimate their abundance. Due to the complexity of metagenomics and the huge volume of sequencing reads of short lengths obtained from the next generation sequencing technologies, the need of efficient statistical tools to accomplish this challenge is increasing. We proposed a two-step procedure to perform functional analysis on a metagenome: mixture model coupled with the adjustment of multiple role assignment, to accurately assign reads to related functional roles by utilizing the SEED classification. Though this research is initiated for the SEED classification, actually the proposed method can be generalized to any type of function annotation system.

Compared to MEGAN and MG-RAST through comprehensive simulation studies, our procedure metaFunction demonstrates more effective in assigning reads to functional roles, thereafter, to subsystems. In the simulation study 1 and 2, the results show that MEGAN cannot assign any read to one of the true functional roles (Figure 4) while in the simulation study 3 and 4, MG-RAST cannot assign any read to one of the true functional roles (plot not shown). This type of phenomenon has never happened to our approach. In addition, the proposed method can correctly assign higher percentage of reads to functional roles than MEGAN does. MEGAN utilizes the best bit-score for assignment. If a read returns with best scores for multiple functions in the BLAST output, then only the first function (alphabetically) is chosen for the assignment. In our method all of them with the same best score are assigned to the read. Different from other existing methods, the proposed method provides confidence intervals for the estimations of the proportions by using bootstrap.

We also applied the proposed method to two real metagenomic datasets and our results generally are consistent with the findings in the previous reports but provide more detailed information. A future work is to integrate the taxonomic analysis and functional analysis, in other words, to consider these two types of issues simultaneously, so that the power can be improved for both taxonomic and functional profiling a metagenomic sample.

## Supporting Information

Figure S1 **Proportions of the detected subsystems (level 3) by MG-RAST and metaFunction for the lake data.** The top 66 subsystems with proportion >0.005 in at least one of samples are listed. The “error” bars represent the 95% confidence interval obtained by bootstrap method. Note: only the proposed approach can provide confidence intervals for the estimations of the proportions.
(TIF)

File S1 Table S1. Number of short reads generated from 10 primary function roles for the studies of 1, 2, and 5. The function names in italic are secondary functions.
Table S2. Number of short reads generated from 10 primary function roles for the studies of 3, 4, and 6. The function names in italic are secondary functions.
(DOCX)
