# Supplementary figures and images for: Statistical Approach of Functional Profiling for a Microbial Community
Source: PLoS One. 2014 Sep 8;9(9):e106588. doi: 10.1371/journal.pone.0106588 (PMC4157783; doi:10.1371/journal.pone.0106588)

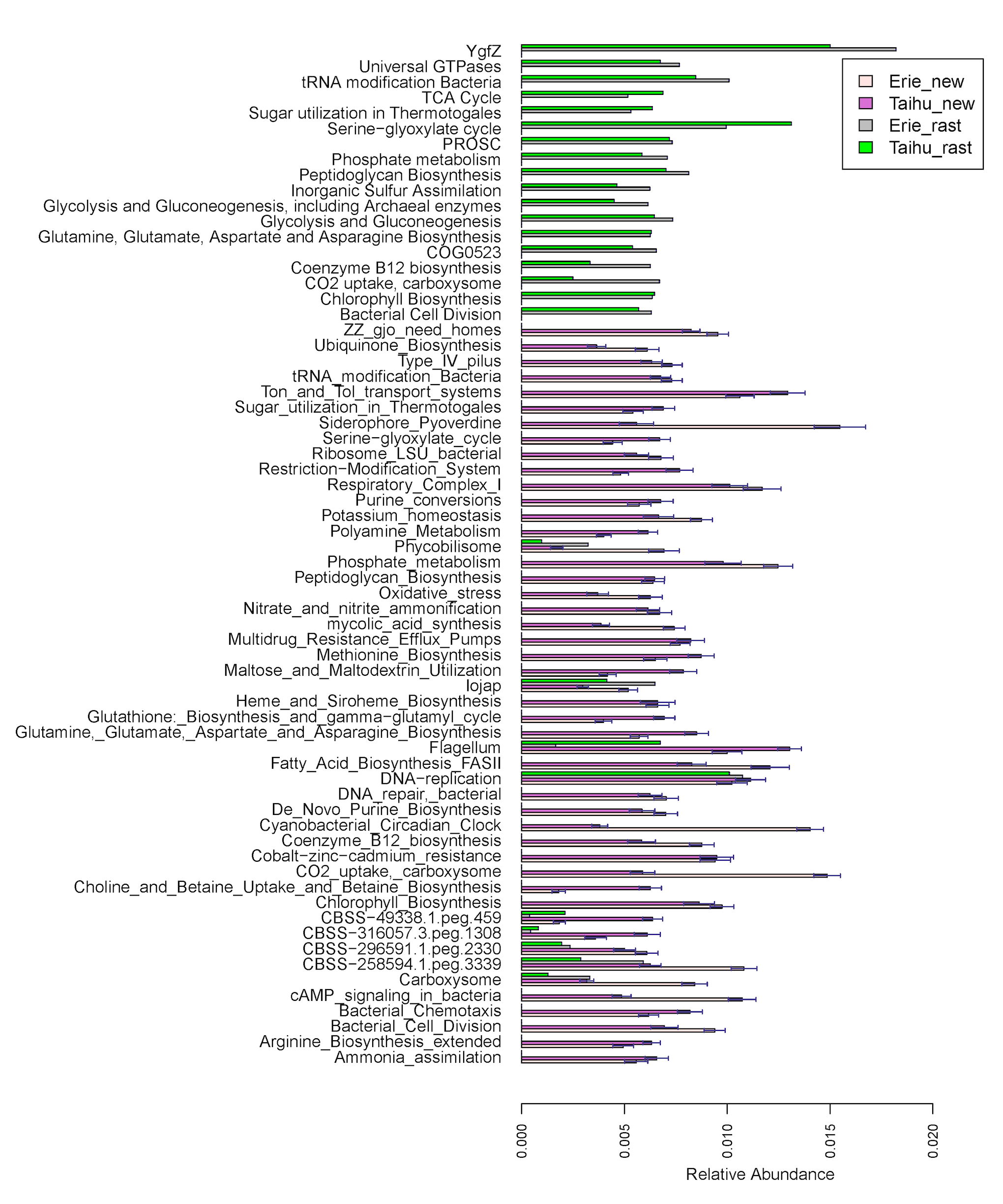

Supplement: Figure S1 — Proportions of the detected subsystems (level 3) by MG-RAST and metaFunction for the lake data. The top 66 subsystems with proportion >0.005 in at least one of samples are listed. The “error” bars represent the 95% confidence interval obtained by bootstrap method. Note: only the proposed approach can provide confidence intervals for the estimations of the proportions. (TIF) [file pone.0106588.s001.tif]
