# Supplementary material for: Statistical Approach of Functional Profiling for a Microbial Community
Source: PLoS One. 2014 Sep 8;9(9):e106588. doi: 10.1371/journal.pone.0106588 (PMC4157783; doi:10.1371/journal.pone.0106588)
Supplement: File S1 — Table S1. Number of short reads generated from 10 primary function roles for the studies of 1, 2, and 5. The function names in italic are secondary functions. Table S2. Number of short reads generated from 10 primary function roles for the studies of 3, 4, and 6. The function names in italic are secondary functions. (DOCX) [file pone.0106588.s002.docx]

**Supplementary**

**Table S1**. Number of short reads generated from 10 primary function roles for the studies of 1, 2, and 5. The function names in italic are secondary functions.

| **Function roles** | **study 1** | **study 2** | **study 5** |
| --- | --- | --- | --- |
| **Diaminopimelate decarboxylase (EC 4.1.1.20)** | 1100 | 1100 | 4500 |
| **Iron-sulfur cluster assembly protein SufB** | 800 | 800 | 3800 |
| **RNA polymerase sigma factor RpoE** | 500 | 800 | 2400 |
| **Polyhydroxyalkanoic acid synthase** | 400 | 600 | 1800 |
| **Virulence factor MviM** | 200 | 400 | 800 |
| **SusC, outer membrane protein involved in starch binding** | 150 | 300 | 700 |
| **Copper resistance protein B** | 150 | 300 | 650 |
| **Ferredoxin reductase** | 150 | 250 | 500 |
| **Signal recognition particle associated protein** | 50 | 100 | 100 |
| **Muconate cycloisomerase (EC 5.5.1.1)** | 50 | 50 | 50 |
| *Aspartokinase (EC 2.7.2.4)* | *24* | *24* | *114* |
| *intein-containing* | *23* | *23* | *130* |
| *At1g63940 homolog* | *14* | *20* | *39* |
| *Protein of unknown function YceH* | *2* | *4* | *10* |
| *putative transport integral membrane protein* | *1* | *1* | *1* |
| **total** | **3614** | **4772** | **15594** |

**Table S2**. Number of short reads generated from 10 primary function roles for the studies of 3, 4, and 6. The function names in italic are secondary functions.

| **Function roles** | **study 3** | **study 4** | **study 6** |
| --- | --- | --- | --- |
| **Single-stranded DNA-binding protein** | 1600 | 1600 | 6950 |
| **membrane c-type cytochrome cy** | 759 | 880 | 1612 |
| **ABC-type protease exporter, membrane fusion protein (MFP) family component PrtE/AprE** | 741 | 870 | 1588 |
| **Arginine/ornithine antiporter ArcD** | 750 | 750 | 3600 |
| **internalin, putative** | 450 | 700 | 2150 |
| **Arginine permease RocE** | 200 | 350 | 750 |
| **Arginine/agmatine antiporter** | 150 | 250 | 500 |
| *invasion plasmid antigen* | *137* | *207* | *611* |
| **Type IV secretion system protein VirD4** | 100 | 200 | 450 |
| **hydrogenase, subunit gamma related protein** | 50 | 100 | 200 |
| **Plasmid partitioning protein ParA** | 50 | 100 | 200 |
| *Type III secretion possible injected virulence protein (YopM)* | *6* | *13* | *51* |
| *putative recombination protein* | *4* | *4* | *24* |
| *putative membrane protein* | *3* | *3* | *4* |
| *cell envelope biogenesis, outer membrane* | *0* | *0* | *2* |
| *membrane protein* | *0* | *0* | *4* |
| **total** | **5000** | **6027** | **18696** |

**Figure S1**. Proportions of the detected subsystems (level 3) by MG-RAST and metaFunction for the lake data. The top 66 subsystems with proportion >0.005 in at least one of samples are listed. The “error” bars represent the 95% confidence interval obtained by bootstrap method. Note: only the proposed approach can provide confidence intervals for the estimations of the proportions.
